# Microdroplet-based one-step RT-PCR for ultrahigh throughput single-cell multiplex gene expression analysis and rare cell detection

**DOI:** 10.1038/s41598-021-86087-4

**Published:** 2021-03-24

**Authors:** Jennifer Ma, Gary Tran, Alwin M. D. Wan, Edmond W. K. Young, Eugenia Kumacheva, Norman N. Iscove, Peter W. Zandstra

**Affiliations:** 1 Institute of Biomedical Engineering, University of Toronto, Toronto, ON M5S 3G9 Canada; 2 Department of Medical Biophysics, University of Toronto, Toronto, ON M5G 1L7 Canada; 3 Department of Mechanical and Industrial Engineering, University of Toronto, Toronto, ON M5S 3G8 Canada; 4 Department of Chemistry, University of Toronto, Toronto, ON M5S 3H6 Canada; 5 Princess Margaret Cancer Centre, University Health Network, Toronto, ON M5G 1L7 Canada; 6 School of Biomedical Engineering, University of British Columbia, 2222 Health Sciences Mall, Vancouver, BC V6T 1Z3 Canada; 7 Michael Smith Laboratories, University of British Columbia, Vancouver, BC V6T 1Z4 Canada

**Keywords:** Reverse transcription polymerase chain reaction, High-throughput screening, Lab-on-a-chip, Computational models, Data processing

## Abstract

Gene expression analysis of individual cells enables characterization of heterogeneous and rare cell populations, yet widespread implementation of existing single-cell gene analysis techniques has been hindered due to limitations in scale, ease, and cost. Here, we present a novel microdroplet-based, one-step reverse-transcriptase polymerase chain reaction (RT-PCR) platform and demonstrate the detection of three targets simultaneously in over 100,000 single cells in a single experiment with a rapid read-out. Our customized reagent cocktail incorporates the bacteriophage T7 gene 2.5 protein to overcome cell lysate-mediated inhibition and allows for one-step RT-PCR of single cells encapsulated in nanoliter droplets. Fluorescent signals indicative of gene expressions are analyzed using a probabilistic deconvolution method to account for ambient RNA and cell doublets and produce single-cell gene signature profiles, as well as predict cell frequencies within heterogeneous samples. We also developed a simulation model to guide experimental design and optimize the accuracy and precision of the assay. Using mixtures of in vitro transcripts and murine cell lines, we demonstrated the detection of single RNA molecules and rare cell populations at a frequency of 0.1%. This low cost, sensitive, and adaptable technique will provide an accessible platform for high throughput single-cell analysis and enable a wide range of research and clinical applications.

## Introduction

Single-cell analysis techniques are critical to distinguish differences between individual cells within seemingly homogeneous populations, such as divergent cell cycle status, cell lineage bias, or other cellular processes. Single-cell analysis techniques can also be applied to the detection of rare cells within heterogeneous cell populations, which would be useful in both basic research and clinical applications^1,2^. Over the last decade, the development of advanced analysis techniques has enabled the study of complex biological systems and phenomena at single-cell resolution. Popular examples include single cell analysis of RNA expression using fluorescence in situ hybridization (FISH)^3–12^, RNA sequencing (scRNA-seq)^13–23^, and reverse-transcription polymerase chain reaction (scRT-PCR).

Despite the advantages of these techniques, they are not exempt from limitations, and can be technically and financially demanding. The sensitivity of FISH and scRNAseq depends on the specific protocols used. While single-molecule FISH (smFISH) is generally regarded as the gold standard for RNA quantification in single cells, the molecular-detection limit of scRNA-seq methods measured using spiked-in transcripts varied over four orders of magnitude^14^. The sensitivity of scRNA-seq is also critically dependent on sequencing depth^14,24^, which scales with the number of cells tested and can significantly drive up experimental cost. This cost (> $400 per 1000 cells^23,25^) can be prohibitive when screening large samples (> 1000 cells) or if frequent sampling is necessary. Moreover, the experimental implementation of FISH and scRNA-seq often requires empirical optimization of multiple sample-specific steps in their procedures, which is time-consuming, labour-intensive, and expensive^26,27^. To improve the sensitivity and throughput of the assays (which would otherwise be confined to thousands of cells), they also require high capital investments (> $150 K) for specialized instruments. Coupling FISH with flow cytometry^11,28^ or fluorescent activated cell sorting^29,30^ has also been explored to increase cellular throughput, concurrently measure gene and protein expression, as well as isolate specific cell populations for downstream analysis. However, these methods are time consuming and have a detection limit of ten copies of transcripts or above. Lastly, high throughput FISH and scRNA-seq assays generate large amounts of complex data, which in turn demand high levels of expertise, complex computational tools, and up to weeks of processing time for bioinformatic analysis^9,31–35^.

RT-PCR is a widely adapted technique due to its low cost, high sensitivity, short turnaround time, and easily customizable assays. However, current single-cell RT-PCR (scRT-PCR) platforms are either limited in cellular throughput or multiplex capability. Microcircuit-based technologies such as the Fluidigm Biomark system allows analysis of up to 192 genes, but the number of single cells captured per run is confined to the hundreds^36–38^. In contrast, microdroplet-based platforms are capable of high-throughput analysis of tens of thousands of cells in a single experiment, but amplification of only one target gene has been shown using this method^39–42^. Moreover, to alleviate cell lysate-mediated inhibition of PCR, all of these techniques employ multiple dilution or reagent addition steps performed in microfluidic devices or manually, which sacrifice sensitivity, limit throughput, and complicate automation of these assays.

As a result, despite the benefit that single-cell analysis can offer in a variety of studies, these state-of-the-art technologies are often restricted to specific applications. Clearly, there remains a need for a low cost, easily adaptable platform that can sample large numbers of single cells and analyze multiple targets simultaneously. Here, we present a microdroplet-based, multiplex RT-PCR platform that is designed for ultrahigh-throughput single-cell analysis of differential gene expression as well as rare cell populations. This technique provides single-cell resolution information with high signal-to-noise ratio and acquisition of hundreds of thousands of data points, which allows the analysis of rare cell populations down to a frequency of at least 0.1%. An in-house reagent mix was developed to overcome amplification inhibition caused by high cell lysate concentration and enabled one-step RT-PCR in nanoliter droplets. The benefit of adding bacteriophage T7 gene 2.5 protein (gp2.5) on relieving cell-lysate mediated inhibition was demonstrated for the first time in this study. To date we have successfully performed simultaneous amplification of three targets in a single encapsulated cell. This multiplex capability makes it a powerful tool for both gene expression analysis and rare cell detection. To enable widespread use in both laboratory and clinical settings, this assay is relatively low cost, easy to perform, readily adaptable for a wide range of applications, and produces reliable results rapidly. The simplicity of our device and protocol allows for future automation of the droplet-based RT-PCR system. This platform will empower research groups by granting them access to single-cell gene expression analysis that would have otherwise been technically and financially difficult.

## Results

### A microfluidic platform for single cell encapsulation and RT-PCR analysis

**Figure 1:**
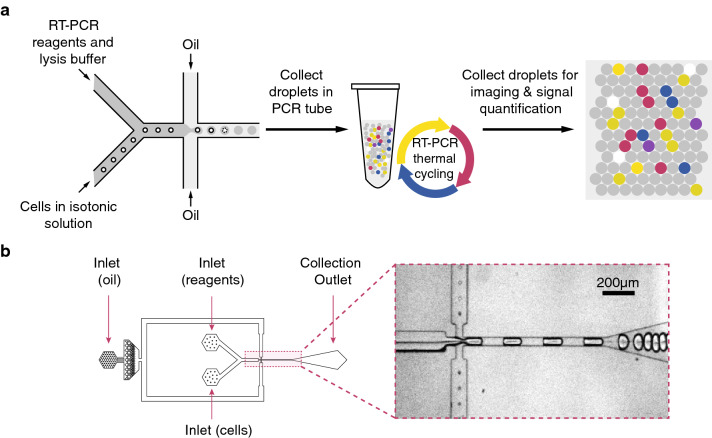
Workflow and devices for droplet-based, single-cell, one-step RT-PCR. (**a**) Process flow of the platform. Cells suspended in an isotonic solution enter a microfluidic device through a channel separate from the RT-PCR reagents and lysis buffer. These channels fuse to a single orifice that injects into a channel met by opposing streams of oil to form monodispersed, 1 nL volume droplets. These droplets are collected in a PCR tube and thermal cycled to generate fluorescent amplification products. The droplets are then deposited in monolayers and analyzed using an automated imaging platform, after which the fluorescent signal in each droplet is quantified. (**b**) Droplet generator design (to scale) and brightfield image of the orifice of the device during droplet formation.

We designed a simple and fully automatable workflow to perform high-throughput single cell RT-PCR. A schematic of the microfluidic (MF) platform is shown in Fig. 1a. Single cells were encapsulated with RT-PCR reagents with lysis buffer in nanoliter (nL) droplets using an MF device. The cell suspension and reagents were delivered through two separate channels, and mixed immediately before the emulsion in oil was formed. The cells rapidly lysed within their respective droplets. The droplets were collected in a PCR tube and subjected to thermal cycling. The droplets, now containing fluorescent amplification products, were loaded into microchambers designed to trap droplets in monolayers for analysis using an automated imaging platform.

Figure 1b shows the design of the MF device. A flow-focusing geometry was chosen due to its ability to form droplets at high capillary numbers and therefore encapsulate at high rates^43^. This feature is beneficial for applications that require large quantities of data points such as rare cell detection. A million droplets of 1 nL volume (approximately 124 µm diameter, see Supplementary Fig. S1 for characterization of droplet diameter in a representative sample) can be generated per hour using a single device.

To quickly image hundreds of thousands of droplets, we used automated fluorescence microscopy. The resulting images were analyzed with the publicly available CellProfiler software. The analysis pipeline identifies droplets by detecting object boundaries in brightfield images and determines their size and shape, which allows for exclusion of droplets that have been broken up or undergone coalescence. The algorithm then measures the average fluorescence signals within each droplet to classify the droplet as positive or negative, either based on a negative control or a clustering algorithm. When microdroplets containing fluorophores were mixed with droplets without fluorophores, the system was capable of enumerating the fluorescent droplets down to ratios of 1 in 10,000 (Supplementary Fig. S1). This demonstrated the capacity of the microscopy system to recognize droplets of various sizes, determine their dimensions, quantify fluorescence intensity within droplets, classify positive droplets, and provide a frequency read-out of rare events.

### Development of multiplex one-step RT-PCR with high cell-lysate tolerance

**Figure 2:**
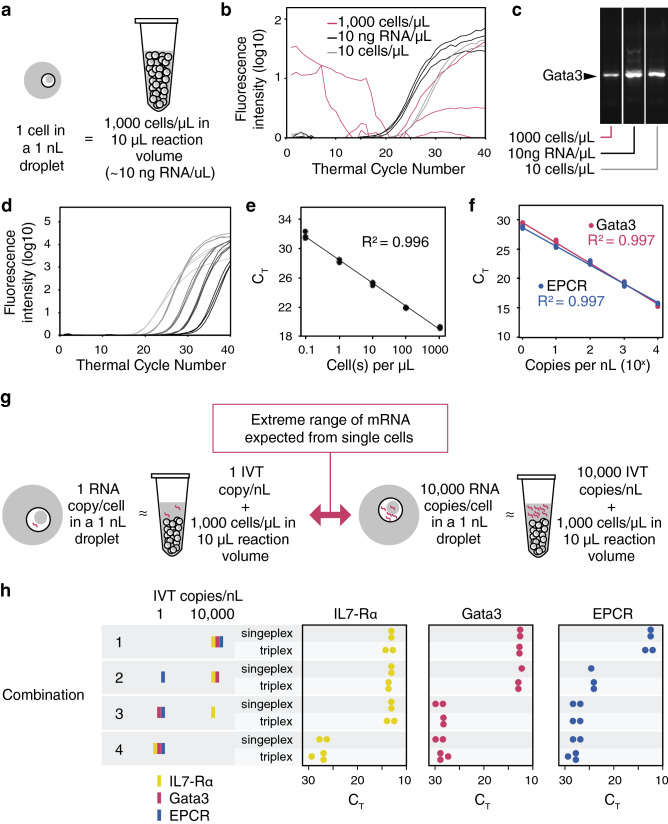
High cell lysate concentrations were shown to be inhibitory to RT-qPCR, thus modifications to current RT-qPCR conditions were required. (**a**) The lysate concentration of a single cell encapsulated in a 1 nL droplet is equivalent to approximately 1000 cells/μL on the scale of conventional RT-qPCR volumes. (**b**,**c**) To demonstrate the inhibitory effect, one-step RT-qPCR was performed to detect Gata3 expression in EL4 cells at 10 and 1000 cells/µL concentration in a 10 µL reaction volume, as well as the amount of total RNA equivalent to 1000 cells/μL (10 ng RNA/μL). Inhibition was observed as either a C_T_ delay or sporadic fluorescence generation with no exponential phase at 1000 cells/µL (**b**). Gel electrophoresis also showed reduced amplification products at higher cell concentration (**c**). See Supplementary Figure S2 for full-length gel image. (**d**) Gata3 mRNA was quantified efficiently on a range of 1 to 10^4^ EL4 cell(s) (shown by darkest to lightest colour plots) in a 10 µL RT-qPCR using our in-house one-step RT-PCR mix with gp2.5. (**e**) Dilutions of cells or (**f**) Gata3 and EPCR IVT doped in constant cell lysate concentrations (1000 cells/µL) were analyzed by RT-qPCR and plotted against their C_T_ values to demonstrate high amplification efficiencies using our in-house one-step RT-PCR mix. (**g**) To emulate the range of mRNA expected from single cells, IL-7Rα, Gata3, and EPCR IVT were doped in constant cell lysate concentrations (1,000 cells/µL) at either 1 or 10,000 copies/nL. (**h**) Using our in-house, multiplex, one-step RT-PCR mix, the three target genes were simultaneously quantified in four different combinations of transcript levels. The difference in C_T_ values obtained using our single and triplex assays were found to be insignificant after 40 thermal cycles (P > 0.1). Each dot represents one technical replicate. Data was analyzed and plotted using JMP (version 15.2.1 www.jmp.com)

Performing RT-qPCR in nano-volume increases the sensitivity of the reaction^44–47^ and reduces the amount of reagents used on a per cell basis. However, previous reports have demonstrated that cell concentrations greater than 200 cells/μL had an inhibitory effect on product yield when performing conventional RT-qPCR and during attempts to downscale the reaction volume in microfluidic devices^39,44,48^. Despite the encapsulation of only a single cell in each 1 nL microdroplet, the cell lysate concentration is equivalent to approximately 1000 cells/μL (Fig. 2a). We found this high cell lysate concentration to inhibit the reaction in conventional RT-qPCR when performed using a sample concentration of 1000 cells/µL. Conventional RT-qPCR reactions were performed with 10 or 1000 cells/µL (Fig. 2b). With the higher cell concentration, generation of fluorescence was chaotic. In contrast, fluorescence developed in the expected cycle-dependent and quantitative pattern in reactions performed with 10 cells/µL. Fluorescence development was similarly quantitative in reactions performed with 10 ng purified total RNA/μL, which is the amount expected in 1000 cells. Agarose gel electrophoresis confirmed that the amount of product generated in the 1000 cells/µL sample was indeed less than that obtained with either 10 cells or purified RNA (Fig. 2c). The results thus confirmed the inhibitory effect of high cell lysate concentration on conventionally performed qRT-PCR reactions.

In an attempt to avoid additional sample dilution or purification steps that would negatively impact the complexity, sensitivity, or efficiency of our platform, we developed an in-house one-step RT-PCR reagent mix that could overcome the lysate-mediated inhibition. To optimize the reaction, RT-qPCR components were tested systematically by altering one component in each reaction. 10,000 cells were added to 10 μL RT-qPCR reactions to simulate the cell lysate concentration in the microdroplets and the effects on C_T_ value, fluorescence plateau, and amplification efficiency were observed. To address inhibition of amplification at high lysate concentration, we explored the effects of adding single-stranded DNA (ssDNA) binding proteins (SSBs) to our RT-qPCR reactions. SSBs have been shown to alleviate inhibitory effects of enzymes and other proteins on PCR reactions^49,50^. The addition of gp2.5 alone was able to partially rescue RT-qPCR performed using either our in-house mix or the CellsDirect One-Step RT-qPCR kit (Invitrogen) from lysate-mediate inhibition (Supplementary Fig. S2). Our resulting RT-qPCR mix was capable of quantifying Gata3 mRNA transcripts directly across a range of 1 to 10,000 EL4 cell(s) in 10 μL RT-qPCRs (Fig. 2d). Based on the slope of the standard curve (α) in Fig. 2e, the reaction efficiency *E* was calculated to be 1.08 (acceptable range = 0.90–1.10)^51^ using the formula

1 $$E={10}^{-1/\alpha }-1$$

Accordingly, gp2.5 was included in the PCR mix for all subsequent experiments.

A broad range of murine Gata3 and EPCR transcript abundances at high cell lysate concentrations were then tested by RT-qPCR (Fig. 2f). Serial tenfold dilutions of Gata3 or EPCR in vitro transcripts (IVT) were added to 10 μL reactions based on the range of transcript concentrations expected in a microdroplet containing a single cell^52,53^, while the cell concentration in the reaction was kept at 1000 cells/μL (using a Gata3 and EPCR negative cell line) to simulate the conditions in the droplets. RT-qPCR analysis performed on the dilution series of the Gata3 and EPCR transcripts yielded R^2^ values greater than 0.99 and efficiencies of 0.94 and 1.06 respectively, indicating that the reactions took place with high efficiency despite the high concentration of cell lysate.

The inability to multiplex has been a critical limitation in current droplet-based RT-PCR platforms. Capability to perform multiplex RT-qPCR would allow for simultaneous detection of multiple surrogate markers in single cells. In single eukaryotic cells, the detectable mRNA transcript abundance in a single cell can range from 1 to 10,000 copies^53^. To mimic the range of mRNA concentrations expected in a 1 nL microdroplet containing a single cell, synthetic mixtures were assembled using four combinations of EPCR, Gata3, and IL7-Rα IVTs at either 1 or 10,000 copies/nL and added into 10 µL RT-qPCRs as template (as illustrated in Fig. 2g and the table in Fig. 2h). The markers Gata3, EPCR, and IL-7Rα were chosen as relevant model genes as they in combination significantly enrich for primitive mouse hematopoietic stem cells (57). Three sets of Taqman probes with different fluorophores were used to quantify the three markers. Into each 10 μL reaction, 10,000 B62c cells were spiked to simulate the amount of lysate in the microdroplets except for combination 4, in which the template composition (low level of IL7-Rα transcripts) conflicted with the high IL7-Rα expression of the B62c cell line. The results of each triplex reaction were compared directly to three individual singleplex reactions performed in parallel to evaluate their performance. The efficiency of the multiplex reaction was determined by comparing the exponential phase of its amplification curve and its C_T_ value to its singleplex counterparts, and the specificity by agarose gel electrophoresis.

High ratio differences in abundance levels between the different genes in the synthetic mixtures resulted in unreliable multi-gene quantification (Supplementary Fig. S3). The amplification of sparse targets was hindered by the presence of abundant targets as they competed for many of the same reagents^54^. This effect was most apparent in IVT combination 3, where IL-7Rα was faithfully amplified but the less abundant Gata3 and EPCR transcripts were not accurately quantified. This IVT combination was, therefore, chosen as the baseline template for optimizing the multiplex reaction by systematically testing the reaction components.

The concentrations of the singleplex RT-qPCR components including binding proteins, salts, dNTPs, and Taq polymerase were reassessed to accommodate simultaneous amplification of three genes, as detailed in Table 1. Primers and Taqman probes were also redesigned to minimize primer-primer interactions across all primer pairs^54,55^. Figure 2h and Supplementary Fig. S4 show the C_T_ values and exponential phases of the triplex reactions using the modified RT-qPCR mix superimposed onto their respective singleplex controls. The difference in C_T_ values obtained using the triplex and singleplex assays were found to be insignificant (two-sample t-test, P > 0.1), demonstrating that the identified multiplex RT-qPCR parameters supported efficient quantification of EPCR, Gata3, and IL7-Rα levels in all 4 IVT combinations despite 10,000-fold differences.

**Table 1:** Summary of modifications to RT-qPCR components for efficient multiplex amplification.

RT-PCR component	Modification	Singleplex	Multiplex
Binding protein gp2.5	The concentration of binding protein was raised to achieve 0.2 µg per pmol of primers and probes to sequester the additional amount of primer pairs and TaqMan probes in multiplex reaction	0.51 µg/µL	0.95 µg/µL
Taq polymerase	The quantity of Taq polymerase was increased for efficient amplification of multiple targets^54,56^	0.3 U/µL	0.6 U/µL
KCl	KCl facilitates annealing of DNA molecules and increase in KCl increases product yield of shorter amplicons^55^	55 mM	90 mM
MgCl_2_	Facilitates annealing of DNA molecules and acts as a cofactor of Taq polymerase^51^	2.0 mM	2.6 mM
dNTP	More dNTPs are required to produce more amplification products. As increasing amounts of dNTPs can bind to Mg^2+^ electrostatically, reduce the amount of Mg^2+^ available and inhibit the reaction^55^, concentrations of MgCl_2_ and dNTPs were tested in a factorial experimental design	400 μM	500 μM
Primers and Taqman probes	Primers and Taqman probes were redesigned and verified by gel electrophoresis to minimize dimer formation and off-target amplification	See Supplementary Table S1 for sequences	See Supplementary Table S1 for sequences

### Simultaneous detection of 3 target genes at single molecule level in nanoliter droplets

**Figure 3:**
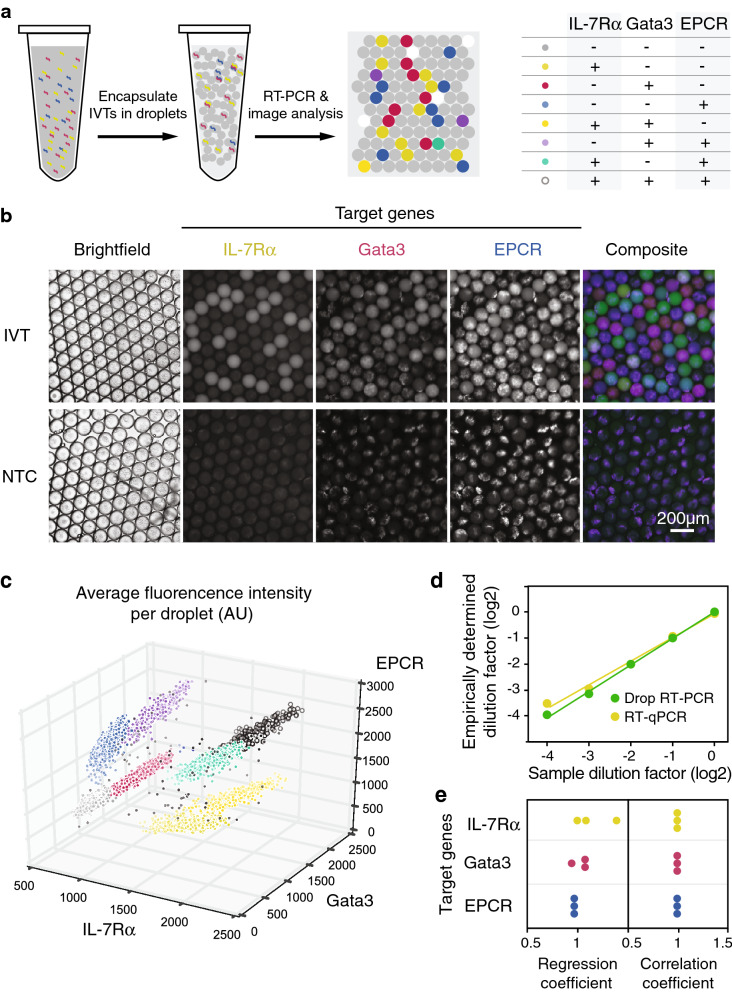
Simultaneous detection of three targets by our droplet RT-PCR platform at single molecule level is demonstrated using limiting dilutions of IVTs. (**a**) Samples containing various dilutions of IL-7Rα, Gata3, and EPCR IVTs were encapsulated for droplet RT-PCR analysis. A substantial increase in fluorescent signal was observed and quantified. The concentration of IVT in each sample was then estimated based on the fraction of droplets with significant increases in fluorescent signal and Poisson statistics. (**b**) Images of droplets containing a mixture of IL-7Rα, Gata3, and EPCR IVTs after amplification targeting the three genes. *NTC* No template control. (**c**) The distribution of fluorescence intensity of each droplet. The colours represent different populations clustered using DBSCAN, and were assigned based on the table in (A) to indicate the presence or absence of the three transcripts in each subpopulation. (**d**) A representative plot showing the correlation between input and estimated concentration of IL-7Rα IVTs using RT-qPCR and droplet RT-PCR. (**e**) The empirically determined dilution factors highly correlated with the sample dilution factors with both regression coefficient and correlation coefficient close to 1 (P < 0.01). Each dot represents one technical replicate. Data was analyzed and plotted using JMP (version 15.2.1 www.jmp.com).

Our next step was to implement the identified multiplex RT-PCR conditions in nanoliter droplets and evaluate the sensitivity of the assay. Limiting dilutions of IL-7Rα, Gata3, and EPCR IVTs were encapsulated with the multiplex RT-PCR mix (Fig. 3a). Compared to the no template control (NTC), substantial increase in fluorescence intensities in the three channels corresponding to the Taqman probes was evident after 50 PCR cycles (Fig. 3b,c). This indicated that the reaction successfully amplified all three transcripts in the droplets. 50 PCR cycles were used to ensure that the reaction in the droplets that contained the target transcripts had reached the plateau phase to maximize the signal-to-noise ratio.

The fluorescence signal was quantified and the droplets were clustered into populations expressing different marker combinations using the density-based spatial clustering algorithm (DBSCAN)^57^ (Fig. 3c), which can identify clusters of arbitrary shapes and does not require prior knowledge of the number of clusters present in each sample. The clusters were labeled as either positive or negative for the three IVTs and the concentrations of the three transcripts in each sample was then estimated utilizing Poisson statistics^58^

2 $$P\left(0\right)={e}^{-\lambda }$$

where *P*(0) is the fraction of droplets that did not express a target gene, and *λ* is the average number of that transcript per droplet. We attained excellent correlation between the empirically determined dilution factors and the sample dilution factors (Fig. 3d,e). This illustrated the ability of the droplet RT-PCR assay to amplify and detect transcripts of 3 target genes simultaneously at single molecule level with high specificity.

### Deconvolution and simulation model for predicting cellular composition of heterogeneous samples

We developed an analytical pipeline that enables deconvolution of single-cell gene signature profile and cellular composition from our droplet RT-PCR assay. The presence or absence of marker genes make up the gene signature of a droplet or a single cell. With three marker genes, there are eight unique gene signatures, as listed in the table in Fig. 2a. A single-cell gene signature profile describes the proportion of single cells in the sample that express each gene signature. As demonstrated in the IVT experiments, a single molecule of RNA can be amplified and produce a positive signal. While this speaks to the sensitivity of our assay, it also means that ambient RNA or cell-free transcripts in the solution released by intact or damaged cells will also be detected. This ambient RNA can be co-encapsulated in droplets with cells, resulting in a false positive signal. Additionally, the encapsulation of cells in droplets is a Poisson process, and the distribution of cells is dependent on the starting cell concentration and droplet volume. Keeping the volume constant, the fraction of droplets that contain more than one cell (multiplets) rises as cell concentration increases. Both ambient RNA and multiplets must be accounted for in order to determine the true gene signature profiles of single cells. The corrected profiles can then be used to estimate the proportions of constituent populations when analyzing heterogeneous samples consisting of multiple cell types.

**Figure 4:**
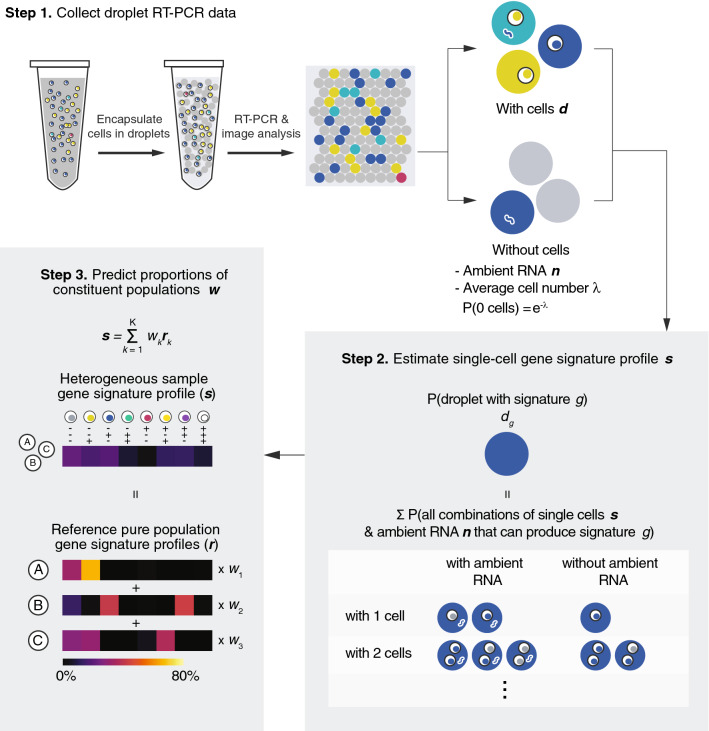
Schematic of deconvolution model to predict single-cell gene signature profiles and the cellular composition of heterogeneous samples. Step 1: Cells are encapsulated and assayed using the droplet RT-PCR platform. The fluorescent signal in every droplet is quantified and the droplet is classified as positive or negative for the presence of cells as well as each target gene. The proportions of droplets that contain cells displaying each gene signature (***d***) are determined. Droplets without cells (empty droplets) inform the probability of observing a certain gene signature in a droplet caused by ambient RNA (***n***). The average number of cells each droplet contains (*λ*) is estimated based on Poisson statistics and the proportions of empty droplets. Step 2: Single-cell gene signature profile of the sample (***s***) is estimated by correcting for ambient RNA and cell doublet effects. Due to the presence of ambient RNA and droplets that contain more than 1 cells, the droplet profile ***d*** does not represent the single-cell gene signature profile of the sample. Considering all the combinations of ambient RNA and cells that can generate a certain signature (up to having 2 cells in each droplet), the proportion of cells expressing each gene signature (***s***) is computed based on the data collected in Step 1. Step 3: The proportions of constituent populations in the heterogeneous sample (***w***) is predicted using the sample (***s***) and reference (***r***) gene signature profiles. Given that the heterogeneous sample is a physical mixture of its constituent cell populations with reference gene signature profiles (***r***) (obtained by assaying pure populations), the composition of the sample mixture (***w***) can be predicted based on the non-negative least squares model using the mixed profile (***s***) obtained from Step 2 and the reference profiles (***r***).

Figure 4 illustrates our analytical pipeline that consists of three steps. Briefly, Step 1 classifies droplets as positive or negative for the presence of cells as well as each target gene based on their fluorescence intensities using the Variational Bayesian Gaussian Mixture with a Dirichlet process prior model^59^. The model assumes that all the data points are generated from a mixture of Gaussian distributions with unknown parameters and does not require a predefined total number of clusters. It then determines the proportions of droplets with cells displaying each gene signature (***d***), the proportions of empty droplets displaying each gene signature caused by ambient RNA (***n***), and the average number of cells each droplet contains (*λ*) estimated based on the proportions of empty droplets.

In Step 2, the single-cell gene signature profile of the sample (***s***) is estimated. ***s***, along with the empirically determined proportions ***d*** and ***n***, are regarded as probability distributions. ***d*** is then modeled as the sum of all possible combinations of ambient RNA and cells (up to having two cells in each droplet to simplify the model) that can generate each signature, allowing the computation of the single-cell profile ***s***.

In Step 3, the proportions of constituent populations in the heterogeneous sample (***w***) are predicted. Given a heterogeneous sample, which is a physical mixture of its constituent cell populations, with gene signature profile ***s,*** its cellular composition ***w*** can be estimated using reference gene signature profiles ***r*** obtained by assaying the pure constituent populations. Based on the non-negative least squares (NNLS) model^60^, the mixture profile ***s*** is modeled as a positively weighted sum of the reference profiles, where weight *w*_*k*_ represents the proportion of reference population *k* within the mixture (assuming that all constituent populations are represented in the reference profiles).

**Figure 5:**
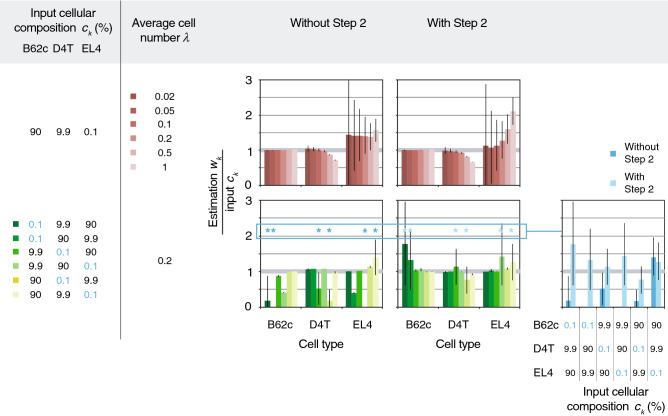
Computer simulation of the droplet assay provides insight into the effects of different input parameters on the accuracy and precision of the assay. Representative results from the simulation illustrating the effects of varying the average cell number per droplet *λ* and the cellular composition ***c***. Three cell lines (B62c, D4T, and EL4) were assayed using the droplet RT-PCR platform to acquire empirical, real-world single-cell gene signature profiles and ambient RNA levels, which were used as input reference profiles and ambient RNA levels (***r*** and ***n***) for the simulation. The bar graphs show predictions made with or without implementing Step 2 of the deconvolution model (means of n = 1000 trials, error bars represent the 95% confidence intervals). As expected, an increase in *λ* (red bar graphs) resulted in higher precision of our predictions, as evidenced by the reduced error bars. The accuracy was improved by incorporating Step 2, which corrected for ambient RNA and duplets, but only when *λ* ≤ 0.2. When varying cellular compositions (green bar graphs), an improvement in accuracy was also observed when Step 2 was applied, whereas the rarest populations (marked by asterisks and plotted separately in the blue bar graph for better comparison) were often undetected without this correction step.

To evaluate our deconvolution model as well as illustrate the effects of various input parameters on the accuracy and precision of our predictions, a simulation of the droplet assay was built to generate thousands of datasets for testing (see Supplementary Fig. S5 for details). We simulated mixtures of three cell lines, B62c (murine pre-B lymphocytes), D4T (murine endothelial cells), and EL4 (murine T lymphocytes), which were assayed using our droplet platform to provide real-world single-cell gene signature profiles and ambient RNA levels as input for our simulations. We also compared the results obtained with and without Step 2 of our analysis pipeline to demonstrate the influence of ambient RNA and multiplets. Representative results from the simulation that reveal the effects of altering average cell number per droplet *λ* and cellular composition of the input cell mixture ***c*** are shown in Fig. 5. The predicted proportions of each cell line fell within the same order of magnitude as the input in all conditions where Step 2 was implemented. It was observed that increasing *λ* boosted the precision of our predictions, as demonstrated by the decrease in confidence intervals. This was due to the higher number of cells “encapsulated” and sampled even though the total number of droplets remained constant (200,000 droplets per experiment). The accuracy of the predictions of the rarest population was improved when Step 2 was incorporated in the analysis, but only when λ was low (≤ 0.2). This result was expected due to the increasing proportion of droplets that contain more than 2 cells in samples where λ was high (0.11% for λ = 0.2, 1.44% for λ = 0.5, 8.02% for λ = 1), which our model did not account for. As for various cellular compositions, we also observed an improvement in accuracy when Step 2 was applied, whereas the rare populations were often undetected without this correction step. As one would expect, the less overlap between the gene signature profiles of the rare (0.1%) and majority (90%) populations, the higher the precision and accuracy attained. Taken together, the simulation results informed the utility and limitations of our assay and analysis pipeline and guided the selection of parameters of our experiments in the next section.

### Single-cell gene expression profiling and rare population detection

**Figure 6:**
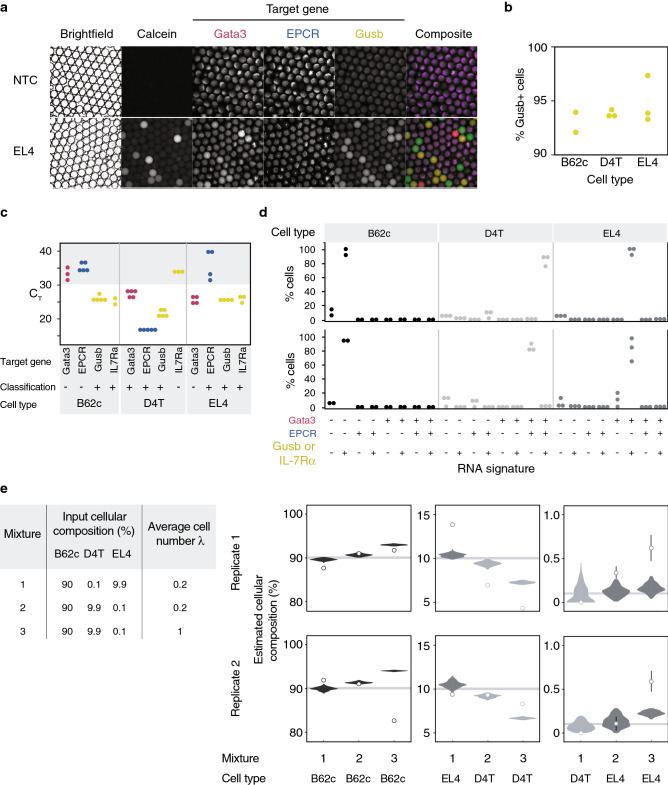
Implementation of droplet RT-PCR for single-cell gene expression profiling and rare cell detection. (**a**) Images of droplets containing EL4 crude cells after amplification targeting Gata3 (red), EPCR (blue), and Gusb (green). A substantial increase in fluorescent signal indicating the expressions of Gata3 and Gusb was observed in the EL4 sample but not in the no template control (NTC). (**b**) Three cell lines (A: B62c, B: D4T, and C: EL4) were assayed using the droplet RT-PCR platform. Detection rates were determined based on the percentage of cells classified as Gusb (housekeeping gene) positive. Each dot represents one technical replicate. (**c**) Population-level gene expression profiles of the three cell lines determined by conventional RT-qPCR. Targets with C_T_ value over 30 are classified as negative. Each dot represents one technical replicate. (**d**) Percentage of single cells expressing each gene signature determined by our droplet RT-PCR assay. Each dot represents one technical replicate. (**e**) Three cell lines were mixed at known ratios and assayed using the droplet RT-PCR platform. The parameters tested are shown in the left table. Results are grouped by the proportions of each cell line in the mixtures and the thick horizontal grey bars indicate the input proportions. The empirical predictions are plotted as dots with error bars representing standard errors, while the results obtained from our computer simulation (100 trials) are overlaid as violin plots.

To test our platform’s capability to perform multiplex single-cell RT-PCR on living cells encapsulated in microdroplets, three murine cell lines with various gene expression profiles, namely B62c, D4T, and EL4, were assayed. No issues were observed when encapsulating the cell lines despite the differences in their cell size and adhesion properties, indicating that our droplet generator can be used on a wide range of cell types. We analyzed the cell lines using the droplet RT-PCR platform based on the expression of murine Gata3, EPCR, and either IL-7Rα or Gusb, a housekeeping gene that is expressed in all three cell lines. Substantial increases in fluorescent amplification products were again observed in all three channels after thermal cycling (Fig. 6a). As expected, Gusb was detected in over 90% of the cells in all cell lines (Fig. 6b), while Gata3, EPCR, and IL-7Rα were present only in subsets of the cell lines, consistent with results obtained by conventional RT-qPCR (Fig. 6c,d). The less than 100% detection rate of Gusb, which concurred with a previous single cell transcriptomic study where Gusb was undetected in 18.6% of cells in the “Mouse Atlas”^61,62^, could be explained by transcriptional bursts of individual genes^63^. Notably, switching out the marker IL-7Rα for Gusb or other genes (data not shown) in the triplex reaction did not require additional optimization of the reaction conditions, suggesting that the assay can be used to target any mRNA targets. This signified the ability of our droplet RT-PCR platform to detect multiple transcript expressions with high specificity and sensitivity in not only purified RNA samples, but also in living cells.

Next, we mixed the three cell lines at predetermined ratios to illustrate the platform’s capability to deconvolve cell populations based on their expression levels. Three different sets of input parameters were selected based on the simulation results (Fig. 6e). Mixture 1 and 2 differed in cellular composition, while Mixture 3 shared the same composition with Mixture 2, but had a higher starting cell concentration thus higher average cell number per droplet. 100,000–200,000 droplets were collected per sample and over 130,000 cells were analyzed per experiment. Conforming to our simulation results, the more abundant the cell type, the more accurately the platform was able to estimate their proportions. The rarest population (0.1%) was undetected in Mixture 1, which was also in agreement with our simulation. In contrast, in Mixture 3, the estimations fell far above the 95% confidence interval of the simulated results. During our analysis, we observed that the increase in cell concentration contributed to an increased background level of the cell tracking dye. This rendered the separation of empty droplets from droplets containing cells more difficult and unreliable, and likely affected the output of the analysis. Mixture 2, on the other hand, consistently produced estimates of the rare population with the highest accuracy, congruent with our simulated results. This demonstrated not only our platform’s ability to enumerate rare cells, but also the power of the simulation for optimizing experimental parameters.

## Discussion

Over the past decade, there has been a surge in technological advancements associated with single-cell transcriptomics. The selection of an appropriate assay for any given application depends on technical considerations, such as throughput, sensitivity, and multiplex capability, which directly affect the information collected, as well as *accessibility* from the perspective of cost, processing time, and any inherent expertise needed to operate and analyze the assay. A survey of existing techniques, considering both technical and accessibility parameters, revealed a wide gap for researchers and clinicians to cross in order to feasibly embark on single-cell studies.

For instance, FISH has the advantage of producing high resolution spatial information, allowing transcript quantification and localization studies within single cells and in the context of tissue structures^3,9,34,40^, and is used as a prognostic and diagnostic tool for diseases such as cancer^2^. scRNA-seq provides extensive information of the transcriptome and is often used for exploratory studies to uncover novel cell populations and their molecular properties^64^. Unfortunately, both of these techniques require expensive equipment and reagents, as well as experienced technicians to perform the experiments and analyze the resulting data^9,25,65^. Neither of these methods have yet to be integrated as standard lab procedures because of the restrictions with respect to the types of applications to which they are most suited.

In contrast, RT-PCR is a well-established, convenient, and economical technique that is broadly used. Advances in high-throughput single-cell capture methods have allowed RT-PCR to be applied to individual cells, but they either suffer from low cellular throughput or are limited to targeting one gene per assay^38,39,42,66–70^. In this paper, we have described an ultrahigh-throughput, multiplex one-step RT-PCR platform that is capable of evaluating gene expression of up to three targets at single-cell resolution. To enable miniaturization of the single-cell assay and massive parallelization, an important breakthrough was identifying an optimal mix of RT-PCR reagents to address the problem of cell lysate-mediated inhibition, a long-standing obstacle to achieving one-step single-cell droplet RT-PCR. This allows robust detection of transcripts in nanoliter volume without downstream manipulation of the droplets, such as dilution or washing. As a result, we do not compromise the sensitivity of the assay due to analyte loss, or complicate the workflow in a way that may negatively impact the potential for system integration and automation.

A critical component of our in-house RT-PCR mix is the bacteriophage T7 gene 2.5 protein. Gp2.5 is a ssDNA binding protein that stimulates DNA synthesis and plays an important role in T7 DNA replication, recombination, and repair^71–74^. Gp2.5 has also been employed as a hot-start strategy by sequestering primers and probes to prevent non-specific binding and amplification at lower temperatures^75^. The addition of SSBs to our RT-PCR was first considered due to their ability to protect RT-PCR reactions from inhibitory cellular contaminants^49,50^. Diverse cellular proteins can interfere with reverse transcription or polymerase reactions, or interact, degrade, and sequester nucleic acids^49,76–78^. Ratnamohan et al.^48^ demonstrated that heat treatment alone was not sufficient to remove cell lysate-mediated inhibition. While proteinase K digestion was able to reduce the effect, subsequent ethanol precipitation that eliminated the denatured proteins and other substances further removed the inhibition. To enable one-step, uncoupled RT-PCR, Chandler et al. experimented with SSBs to relieve inhibition of PCR amplification by residual reverse transcriptase, which was shown to interact directly with the specific primer-template complex^49^. Including SSBs during, but not after, the RT phase, increased the RT-PCR product yield by almost fivefold. This suggested that first-strand cDNA synthesis was more efficient in the presence of SSBs. SSBs such as gp2.5 are known to disrupt the secondary structure of ssDNA^79^ and may hold the nucleotides in a favourable conformation for pairing with complementary nucleotides^80^. A recent study also showed that gp2.5 can facilitate fast template-primer hybridization, increasing the hybridization rate of complementary ssDNA strands by 36-fold^81^. Furthermore, given their high affinity to ssDNA strands^79,82^, binding proteins may displace inhibitory substances from the templates and primers, allowing them to participate in RT. These suggest a potential use of gp2.5 as a protectant and facilitator of template-primer hybridization in high cell lysate concentrations, where PCR inhibitors are abundant.

Additionally, single transcripts of IVT and ambient mRNA were detected at limiting dilutions using our droplet platform even in the presence of high cell lysate concentrations. This demonstrates high sensitivity of the PCR chemistry capable of detecting low abundance transcripts in the lysate. However, it is noted that the procedures used here for disrupting the cells do not ensure that every expressed transcript in a cell is accessible. For example, transcripts located within the nucleus may not be liberated, and cytoplasmic transcripts could be physically unavailable for other reasons. Future studies that explore transcript availability and compare our platform with others such as smFISH will allow better insight into the detection limit for endogenous mRNA.

We are also the first to demonstrate multiplex RT-PCR of single cells in nanoliter droplets, greatly enhancing the capability of our platform to perform gene expression profiling of single cells and identify highly specific populations of cells. Our assay can potentially be engineered to detect more genes via further optimization and approaches such as amplitude modulation, where the relative TaqMan probe concentrations amongst targets are varied to yield distinctive PCR curves^83,84^. Since PCR primers and probes can be designed for any DNA and RNA targets, this technique can be readily adapted to analyze any cell population of interest.

Our technique does not rely on solid-phase RNA capture, ensuring high sensitivity and cell capture rate. This makes it suitable for assaying low-input samples such as clinical specimens with minimal cell loss. Potential applications include dissecting the composition of a tumor sample for diagnosis and prognosis. We have also demonstrated the platform's ability to handle large samples that consist of hundreds of thousands of cells. The current flow rate can be raised by at least fivefold without causing cell damage^85^, and multiple devices can operate in parallel to further improve the speed of droplet generation. Additionally, automated imaging analysis enables rapid signal quantification and visualization of the samples, which can be manually inspected to ensure correct interpretation of the results. Combined with the high throughput, this feature is especially beneficial for applications that have low tolerance for false positives, such as detecting and characterizing rare cell types based on their gene expression profiles. As demonstrated in this study, our platform can detect rare populations at a frequency of 0.1%, which is equivalent to the number of blood stem cells found in primitive populations derived from umbilical cord blood^86^. An exciting application of our platform would be for enumerating these highly regenerative cells for therapeutic and research purposes. Other potential applications include assessing minimal residual disease, detection of rare cells with viral infection or genetic abnormalities, and detection of fetal cells in maternal blood for non-invasive prenatal diagnosis^1^. Similar to the calcein dye used in this study that tagged live cells, other biomolecules such as proteins can also be fluorescently labeled prior to cell encapsulation and visualized in the droplets after cell lysis. This allows analysis of the co-expression of different transcripts and biomolecules, whereas sorting for droplets with specific biomolecules can potentially be built into the microfluidic device. Another future development of our droplet system is to implement real-time imaging during the RT-PCR cycles to allow for transcript quantification. The ability to reliably quantify gene expression in hundreds of thousands of single cells would be highly valuable.

Our computational tools for simulating, predicting, and analyzing the results of our assay are also proven to be crucial for maximizing the accuracy of its readout, especially when applied to rare population detection. Our simulation model can be used to guide experimental design, including the selection of surrogate markers, sample size, and cell concentration, to achieve optimal results. Our deconvolution pipeline corrects for background noise from ambient RNA as well as doublets in the samples, which are issues commonly seen in droplet RT-PCR techniques but seldom addressed. The former contributes to a robust assay that is less sensitive to the quality of the cell sample. The latter allows us to achieve higher cellular throughput without increasing the number of droplets, therefore reduces reagent and time consumption without compromising the accuracy of our assay. This may be further improved by expanding our model to account for a higher number of multiplets. Additionally, our current model relies on pre-specified reference gene signature profiles that accurately portraits the constituent populations. More advanced deconvolution algorithms that can accommodate populations that are not included in the reference populations^87^, or perturbations due to microenvironmental or developmental effects that alter the gene expression of the constituent populations^88^ could improve the accuracy of the prediction and broaden the application of the assay. Our analysis pipeline requires minimal user supervision except for labeling identified clusters of populations, which can potentially be automated as well to further enhance usability.

At roughly $3 per 1000 cells (see Supplementary Table S3 for cost breakdown), our experimental cost falls within an affordable range that allows routine measurements. Our setup does not require expensive and highly specialized instrumentation and software, and can be assembled using pieces of apparatus readily available in a standard biology lab. The key to further drive down cost in the future such that millions or more cells can be assayed will be to decrease reagent consumption by reducing droplet size, increasing droplet stability, and improving encapsulation efficiency through new technologies that can enforce allocation of a single cell to each droplet in place of Poisson distribution.

In summary, our unique platform takes advantage of the miniaturization, compartmentalization, and precise liquid handling capabilities of the ultrahigh-throughput microdroplet system to develop an automatable, low-cost, and highly sensitive assay. As such, this novel technique provides a better single-cell analysis solution for many applications than other existing platforms, and has a high potential to be implemented in both clinical and research settings for rapid and direct evaluation of single-cell gene expression.

## Materials and methods

### Microfluidic device fabrication

The MF droplet generators were fabricated by soft lithography following standard protocols (SI Materials and Methods). The imaging chip with microchambers was fabricated from 1.5 mm thick sheets of poly(methyl methacrylate) (PMMA) (#8560K173, McMaster-Carr, Elmhurst, IL, USA). All parts were fabricated with computer numerical control (CNC) milling using a Tormach PCNC 770 vertical milling machine (Tormach Inc., Waunakee, WI). Microchannels and microfeatures (patterned into devices) were modelled with SolidWorks (Dassault Systemes, Velizy-Villacoublay, France). The CNC program was created with SprutCAM (SprutCAM, Naberezhnye Chelny, Russia). Devices were milled using a 1/32″ (794 µm) 4 flute carbide endmill (#89318919, MSC Industrial Supply Co., Melville, NY, USA), using procedures previously described^89^. Milled devices were sealed using solvent bonding procedures previously described^90^.

### Droplet generation

Cell suspension and RT-PCR reagents were loaded into 0.5 mL syringes (B305620, BD) and injected at an equal flow rate of 0.5 mL/h into a droplet generator through polyethylene tubing (427405, BD) using a syringe pump (70–4505, Harvard Apparatus). QX200 Droplet Generation Oil for EvaGreen Assays (1864006, Bio-Rad) was used for the oil phase and loaded into a 3 mL syringe. The flow rate (~ 2 mL/h) of the fluorinated oil was adjusted and controlled using a syringe pump to achieve the desired droplet size of 124 µm diameter. The droplet generation process was monitored using an inverted microscope (AE2000, Motic). The emulsions were collected through the outlet and polyethylene tubing into 1.5 mL microcentrifuge tubes (MCT-150-A, Axygen) before transferring to 0.2 mL PCR tubes (PCR-02-C, Axygen) for thermal cycling.

### One-step RT-PCR and primer design

RT-PCR reagents were assembled on ice from one-time use aliquots right before the experiments. Either the CellsDirect One Step RT-qPCR kit (Invitrogen) or an in-house RT-qPCR mix was used. See Table 2 for the composition of the simplex and multiplex in-house reaction mixtures. Either the forward or reverse primer of each target gene was designed to span an exon-exon junction to avoid amplifying genomic DNA. TaqMan hydrolysis probes were used for quantification of product generation. PREMIER Biosoft Beacon Designer 8.0 was used as the first step to design the TaqMan assays with the following criteria:

- Primers: 18–25 bp; melting temperature T_m_ = 56 ± 4 °C; amplicon length = 80–200 bp; exon junction spanning.
- Taqman probes: 18–30 bp; T_m_ = (T_m_ of primers) +  ~ 10 °C.

**Table 2:** Formulation of the simplex and multiplex RT-PCR reagent mixes.

Reagents	Unit	Conventional RT-qPCR			Droplet RT-PCR	
		Simplex (before optimization)	Simplex	Multiplex	IVT multiplex	Crude cell multiplex
TrisHCl, pH 8.0	mM	10	30	30	30	30
KCl	mM	55	55	90	70	70
MgCl_2_	mM	2	2	2.5	2.5	2.5
SUPERase•In RNase Inhibitor (Invitrogen)	U/μL	1	1	1	1	1
Taq polymerase (produced in Iscove lab)	U/μL	0.1	0.3	0.6	0.6	0.6
HotStart-IT Binding Protein (Thermo Scientific)	ng/μL	510	510	950	950	950
dNTPs	mM	0.4	0.4	0.5	0.5	0.5
Forward & reverse primers (Sigma-Aldrich)	uM	0.3	0.9	0.6	0.6	0.6
TaqMan MGB Probe (Applied Biosystems)	nM	250	350	350	350	350
Nonidet P-40	% vol	0.01	0.01	0.01	0.01	0.14
Pluronic F-68 Non-ionic Surfactant (Gibco)	% vol	0	0	0	0.05	0.2
SuperScript III reverse transcriptase (Invitrogen)	U/μL	1	1	1	1	1
Bovine serum albumin (Roche)	ng/μL	150	450	450	1380	1380
DTT (Invitrogen)	mM	1	1	1	1	1
ROX passive reference dye	nM	50	50	50	0	0

TaqMan probes were designed with a higher T_m_ to ensure that they hybridize to their targets before the primers. Beacon Designer and NetPrimer were used to identify regions of the primers prone to form primer-dimers, and particular attention was paid to avoid GC-rich complementary regions. Assays were examined for unintended targets using NCBI Primer-BLAST and known SNPs were avoided using NCBI dbSNP. The sequences of the primers and probes directed against murine mRNAs are listed in Supplementary Table S1. IVTs were prepared using the MEGAscript T7 Transcription Kit (Invitrogen) and purified using TRIzol Reagent (Invitrogen) (see Supplementary Materials and Methods for detailed protocol and Supplementary Table S2 for primer sequences).

The samples were combined with the reagents either in 384-well PCR plates (for conventional RT-qPCR) or in droplets collected in PCR tubes (for single-cell RT-PCR). They were then subjected to the following thermal cycles: 55 °C for 30 min, 95 °C for 2 min, and 40 cycles of 95 °C for 15 s, 55 °C or 58 °C for 30 s, and 72 °C for 30 s. RT-qPCR was performed using an Applied Biosystems 7900HT instrument, and droplet RT-PCR was performed with a Biometra T-Personal Thermal Cycler.

### Cell culture and fluorescence staining

EL4 and D4T cells were cultured in IMDM (Gibco) supplemented with 10% FBS (Gibco). B62c cells were cultured in IMDM (Gibco) supplemented with 10% FBS (Gibco) and 0.63%(v/v) alpha-thioglycerol. Conditioned media with IL-7 was added directly to B62c culture every 3 days at a ratio of 1:200 total volume. The EL4, D4T, and B62c cell lines were kind gifts from the Mak lab, the Iscove lab, and the Paige lab in Toronto.

To stain the cells with eBioscience Calcein Violet 450 AM Viability Dye (Invitrogen), D4T cells were first dissociated using 0.25% Trypsin–EDTA (Gibco) at 37 °C for 2 min and then suspended in culture medium. Each cell line was resuspended at a concentration of 10^6^ cells/mL in PBS with 100 μM of Calcein Violet after centrifugation (200*g* for 5 min for EL4 and D4T cells, 450 *g* for 10 min for B62c cells) and incubated at 37 °C for 20 min, protected from light. 5 times the PBS volume of culture medium was then added to the cells and incubated at 37 °C for 5 min. The cells were pelleted by centrifugation and resuspended in fresh culture medium. After incubating for 30 min at 37 °C, the cells were centrifuged and resuspended in a 2.2% (w/w) glycine solution with 0.1 µg/mL Ambion RNase A (Invitrogen). The concentration and mixture of different cell types were adjusted based on the experiments they were prepared for. The cells were then incubated on ice for 3 h to reduce the amount of ambient RNA prior to encapsulation.

### High-throughput droplet imaging and analysis

Droplets were loaded into microchambers on an imaging chip using a micropipette. The chips were then placed on a slide holder for automated imaging using the Cellomics Arrayscan VTI platform (Thermo Scientific). The slides were imaged with a 5X widefield objective in brightfield and 4 fluorescence channels that corresponded to the Calcein dye and Taqman probes, rendering 64 field images per microchamber per channel. These field images were tiled into one image per chamber per channel using an in-house Python script and analyzed in CellProfiler to quantify the size and total fluorescence intensities of the droplets.

### Analytical pipeline and deconvolution model

Our models are implemented in Julia and are available online at https://gitlab.com/ stemcellbioengineering/droplet-rtpcr along with the parameters tested. In the following descriptions, variables are in italics, vectors are in bold, constants are in uppercase. Our models were built with the following assumptions:

- Empty droplets can be distinguished from droplets with cells.
- The proportion of droplets with more than two cells is negligible. The efficiency of the reaction in each droplet is not affected by the number of cells it contains.
- Cells and ambient RNA in a droplet can combine to produce new signatures. The possible signatures of a droplet can range from 1 (–-) to G = 2^3^ (+ + +), where each bit represents a marker, + means positive and—means negative.
- All constituent populations in heterogeneous cell mixtures are represented in the reference profiles. The reference profiles are obtained by assaying individual reference cell types with the droplet RT-PCR platform and provided as input to deconvolve the cellular composition of cell mixtures.

The model imports data exported by Cellprofiler, which consists of the area and fluorescence intensities of each droplet. The upper and lower limits for droplet size can be assigned to discard droplets that are either too big or too small. The Variational Bayesian Gaussian Mixture algorithm with a Dirichlet process prior model (DPGMM) from the scikit-learn Python package^91^ is used to cluster the droplets based on their fluorescence intensity from the Calcein dye. The clusters are then manually assigned as positive or negative for Calcein to determine the presence of cells in the droplet. The average number of cells per droplet *λ* is estimated based on the proportion of empty droplets and Poisson statistics using Eq. (2).

DPGMM is applied to cluster droplets based on their fluorescence intensities from the 3 Taqman probes, and the clusters are assigned as either positive or negative for each target gene. The proportions of droplets with cells that display each gene signature are determined, where ***d*** is a vector of length G and *d*_*g*_ is the proportion of droplets that contain cells and display the gene signature *g*. The probabilities of observing different gene signatures caused by ambient RNA alone are also estimated, where ***n*** is a vector of length G, and *n*_*g*_ is the proportion of empty droplets in the sample that display the gene signature *g*.

***d*** is then modeled as the sum of all possible combinations of ambient RNA and cells that can generate each signature. The single-cell gene expression profile, where ***s*** is a vector of length G, and *s*_*g*_ is the proportion of single cells in the sample that express the gene signature *g*, is solved iteratively from the following system of equations:

3 $${d}_{1}={p}_{1}{n}_{1}{s}_{1}+{p}_{2}{n}_{1}{{s}_{1}}^{2}$$

4 $${d}_{2}={p}_{1}\left[{n}_{2}{s}_{1}+\left({n}_{1}+{n}_{2}\right){s}_{2}\right]+{p}_{2}\left[{n}_{2}{{s}_{1}}^{2}+\left({n}_{1}+{n}_{2}\right)\left({{s}_{2}}^{2}+{s}_{1}{s}_{2}\right)\right]$$

5 $${d}_{3}={p}_{1}\left[{n}_{3}{s}_{1}+\left({n}_{1}+{n}_{3}\right){s}_{3}\right]+{p}_{2}\left[{n}_{3}{{s}_{1}}^{2}+\left({n}_{1}+{n}_{3}\right)\left({{s}_{3}}^{2}+{s}_{1}{s}_{3}\right)\right]$$

6 $${d}_{4}={p}_{1}\left[{n}_{4}{s}_{1}+\left({n}_{3}+{n}_{4}\right){s}_{2}+\left({n}_{2}+{n}_{4}\right){s}_{3}+\left({n}_{1}+{n}_{2}+{n}_{3}+{n}_{4}\right){s}_{4}\right]+{p}_{2}\left\{{n}_{4}{{s}_{1}}^{2}+\left({n}_{3}+{n}_{4}\right)\left({{s}_{2}}^{2}+{s}_{1}{s}_{2}\right)+\left({n}_{2}+{n}_{4}\right)\left({{s}_{3}}^{2}+{s}_{1}{s}_{3}\right)+\left({n}_{1}+{n}_{2}+{n}_{3}+{n}_{4}\right)\left[{{s}_{4}}^{2}+\left({s}_{1}+{s}_{2}+{s}_{3}\right){s}_{4}+{s}_{2}{s}_{3}\right]\right\}$$

7 $${d}_{5}={p}_{1}\left[{n}_{5}{s}_{1}+\left({n}_{1}+{n}_{5}\right){s}_{5}\right]+{p}_{2}\left[{n}_{5}{{s}_{1}}^{2}+\left({n}_{1}+{n}_{5}\right)\left({{s}_{5}}^{2}+{s}_{1}{s}_{5}\right)\right]$$

8 $${d}_{6}={p}_{1}\left[{n}_{6}{s}_{1}+\left({n}_{5}+{n}_{6}\right){s}_{2}+\left({n}_{2}+{n}_{6}\right){s}_{5}+\left({n}_{1}+{n}_{2}+{n}_{5}+{n}_{6}\right){s}_{6}\right]+{p}_{2}\left\{{n}_{6}{{s}_{1}}^{2}+\left({n}_{5}+{n}_{6}\right)\left({{s}_{2}}^{2}+{s}_{1}{s}_{2}\right)+\left({n}_{2}+{n}_{6}\right)\left({{s}_{5}}^{2}+{s}_{1}{s}_{5}\right)+\left({n}_{1}+{n}_{2}+{n}_{5}+{n}_{6}\right)\left[{{s}_{6}}^{2}+\left({s}_{1}+{s}_{2}+{s}_{5}\right){s}_{6}+{s}_{2}{s}_{5}\right]\right\}$$

9 $${d}_{7}={p}_{1}\left[{n}_{7}{s}_{1}+\left({n}_{5}+{n}_{7}\right){s}_{3}+\left({n}_{3}+{n}_{7}\right){s}_{5}+\left({n}_{1}+{n}_{3}+{n}_{5}+{n}_{7}\right){s}_{7}\right]+{p}_{2}\left\{{n}_{7}{{s}_{1}}^{2}+\left({n}_{5}+{n}_{7}\right)\left({{s}_{3}}^{2}+{s}_{1}{s}_{3}\right)+\left({n}_{3}+{n}_{7}\right)\left({{s}_{5}}^{2}+{s}_{1}{s}_{5}\right)+\left({n}_{1}+{n}_{3}+{n}_{5}+{n}_{7}\right)\left[{{s}_{7}}^{2}+\left({s}_{1}+{s}_{3}+{s}_{5}\right){s}_{7}+{s}_{3}{s}_{5}\right]\right\}$$

10 $${d}_{8}={p}_{1}\left[{n}_{8}{s}_{1}+\left({n}_{7}+{n}_{8}\right){s}_{2}+\left({n}_{6}+{n}_{8}\right){s}_{3}+\left({n}_{5}+{n}_{6}+{n}_{7}+{n}_{8}\right){s}_{4}+\left({n}_{4}+{n}_{8}\right){s}_{5}+\left({n}_{3}+{n}_{4}+{n}_{7}+{n}_{8}\right){s}_{6}+\left({n}_{2}+{n}_{4}+{n}_{6}+{n}_{8}\right){s}_{7}+{s}_{8}\right]+{p}_{2}\left\{{n}_{8}{{s}_{1}}^{2}+\left({n}_{7}+{n}_{8}\right)\left({{s}_{2}}^{2}+{s}_{1}{s}_{2}\right)+\left({n}_{6}+{n}_{8}\right)\left({{s}_{3}}^{2}+{s}_{1}{s}_{3}\right)+\left({n}_{5}+{n}_{6}+{n}_{7}+{n}_{8}\right)\left[{{s}_{4}}^{2}+{s}_{4}\left({s}_{1}+{s}_{2}+{s}_{3}\right)+{s}_{2}{s}_{3}\right]+\left({n}_{4}+{n}_{8}\right)\left({{s}_{5}}^{2}+{s}_{1}{s}_{5}\right)+\left({n}_{3}+{n}_{4}+{n}_{7}+{n}_{8}\right)\left[{{s}_{6}}^{2}+{s}_{6}\left({s}_{1}+{s}_{2}+{s}_{5}\right)+{s}_{2}{s}_{5}\right]+\left({n}_{2}+{n}_{4}+{n}_{6}+{n}_{8}\right)\left[{{s}_{7}}^{2}+{s}_{7}\left({s}_{1}+{s}_{3}+{s}_{5}\right)+{s}_{3}{s}_{5}\right]+{s}_{2}{s}_{7}+{s}_{3}{s}_{6}+{s}_{4}\left({s}_{5}+{s}_{6}+{s}_{7}\right)+{s}_{6}{s}_{7}+{s}_{8}\right\}$$

where and *p*_*1*_ and *p*_*2*_ represent the normalized probability of capturing 1 and 2 cells in a droplet that contain cells respectively:

11 $${p}_{x}=\frac{P\left(x\, cell\right)}{P\left(1\, cell\right)+P(2\, cells)}$$

and *P(x cells)* represents the probability of capturing *x* cells in a droplet based on Poisson distribution given *λ*:

12 $$P\left(x\, cells\right)=\frac{{\lambda }^{x}{e}^{-\lambda }}{x!}$$

The single-cell profile of the heterogeneous sample ***s*** is then modeled as a linear combination of signature profiles of the reference populations, ***r***_*k*_, weighted by mixture proportions ***w***:

13 $${\varvec{s}}=\sum_{k=1}^{K}{w}_{k}{{\varvec{r}}}_{k}$$

where weight *w*_*k*_ represents the proportion of reference population *k* in the sample. ***r***_*k*_ is a vector of length G and *r*_*k,g*_ is the proportion of cell type *k* that expresses the gene signature *g*. The nnls() function from the NNLS package in Julia, which implements the non-negative least squares solver from^60^, is used to estimate the optimal non-negative values of *w*_*k*_.

### Simulation model

The simulation model was designed to emulate the droplet RT-PCR experiment. Input parameters include the total number of droplets in each experiment, the average number of cells per droplet, as well as the signature profiles and concentration of each cell type. The simulation can be summarized as follows:

- The total number of droplets deposited per trial is *D*.
- The number of cells in any given droplet follows a Poisson distribution with a mean (average number of cells per droplet) of *λ* as predicted by Eq. (11).
- Cells of type *k* have a concentration of *c*_*k*_ in a well-mixed population. Cells are drawn randomly with probability equal to their concentration.
- Each cell type is characterized by two sets of signature profiles. ***r***_*k*_ is a vector of length G and *r*_*k,g*_ is the proportion of cell type *k* that expresses the gene signature *g*. Cell signatures are assigned randomly with probability equal to its proportion. ***n***_*k*_ is a vector of length G, and *n*_*k,g*_ is the proportion of empty droplets that display the gene signature *g* when assaying cell type *k*.
- The levels of ambient RNA of the sample ***m*** is modeled as a linear combination of the ambient RNA levels of the constituent cell populations, ***n***_*k*_, weighted by mixture proportions ***c***:
  14 $${\varvec{m}}=\sum_{k=1}^{K}{c}_{k}{{\varvec{n}}}_{k}$$

Ambient RNA signatures are assigned randomly with probability equal to its proportion.

- Cells and ambient RNA in a droplet are combined to produce the signature of the droplet.
- Once results are obtained from simulations, the cellular composition of the sample ***w*** is estimated using our deconvolution model described above and compared with the initial input concentrations ***c***.
- The experiment is run thousands of times with different random seeds to numerically obtain key statistics of the estimated cellular composition of the sample.

## Supplementary Information

Supplementary Information

## Data Availability

The datasets generated during and/or analysed during the current study are available from the corresponding author on reasonable request.
