# Supplementary material for: Microdroplet-based one-step RT-PCR for ultrahigh throughput single-cell multiplex gene expression analysis and rare cell detection
Source: Sci Rep. 2021 Mar 24;11:6777. doi: 10.1038/s41598-021-86087-4 (PMC7990930; doi:10.1038/s41598-021-86087-4)
Supplement: Supplementary file 1 — Supplementary Information [file 41598_2021_86087_MOESM1_ESM.pdf]

Supplementary Information

Microdroplet-based one-step RT-PCR for ultrahigh throughput single-cell multiplex gene expression analysis and rare cell detection

Jennifer Ma[a], Gary Tran[b], Alwin M. D. Wan[c], Edmond W. K. Young[a,c], Eugenia Kumacheva[a,c,d], Norman N. Iscove[b,e,f,1], Peter W. Zandstra[g,h,1,*]

[a]Institute of Biomedical Engineering, University of Toronto, Toronto, ON M5S 3G9, Canada
[b]Department of Medical Biophysics, University of Toronto, Toronto, Ontario M5G 1L7, Canada
[c]Department of Mechanical & Industrial Engineering, University of Toronto, Toronto, Ontario M5S 3G8 Canada
[d]Department of Chemistry, University of Toronto, Toronto, Ontario M5S 3H6, Canada
[e]Department of Chemical Engineering and Applied Chemistry, University of Toronto, Toronto, Ontario M5S 3E5, Canada
[f]Princess Margaret Cancer Centre, University Health Network, Toronto, Ontario M5G 1L7, Canada
[g]School of Biomedical Engineering, University of British Columbia, Vancouver, British Columbia V6T 1Z3, Canada.
[h]Michael Smith Laboratories, University of British Columbia, Vancouver, British Columbia V6T 1Z4, Canada.

[1]These authors share senior authorship

Corresponding author: Peter W. Zandstra

Address: 2222 Health Sciences Mall, Vancouver BC, V6T 1Z3 Canada

Phone: 604 822 2694

Email: peter.zandstra@ubc.ca

Supplementary Figures

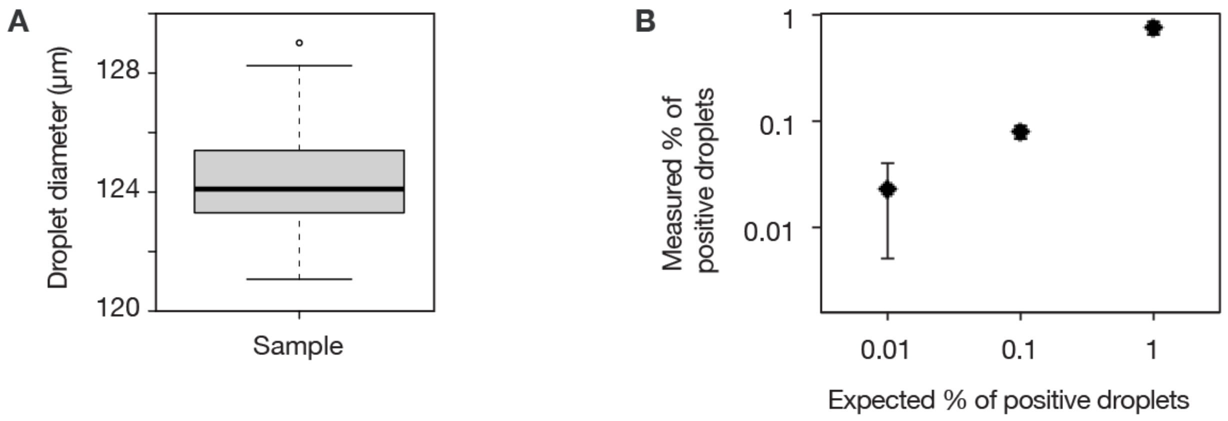

Figure S1. Droplet characterization and rare droplet enumeration.

(A) Diameter of droplets collected from one experiment to ensure droplet diameter fell around 124 µm. Small samples of droplets were collected on a glass slide during droplet generation and imaged using an inverted microscope. The diameter of the droplets were determined using ImageJ. Centerline of the box plot shows the median; box limits indicate the 25th and 75th percentiles as determined by R software; whiskers extend 1.5 times the interquartile range from the 25th and 75th percentiles, outlier is represented by a dot. n = 68 sample points.

(B) Our image analysis platform was able to detect and enumerate microdroplets with fluorescence signal down to ratios of 1 in 10,000. Microdroplets were generated using pure PBS or fluorophore conjugated antibodies diluted in PBS. The fluorescent droplets were mixed with droplets without fluorophores at ratios of 1 in 100, 1,000, and 10,000. The mixtures were then imaged using automated fluorescence microscopy to estimate the proportions of fluorescence positive droplets.

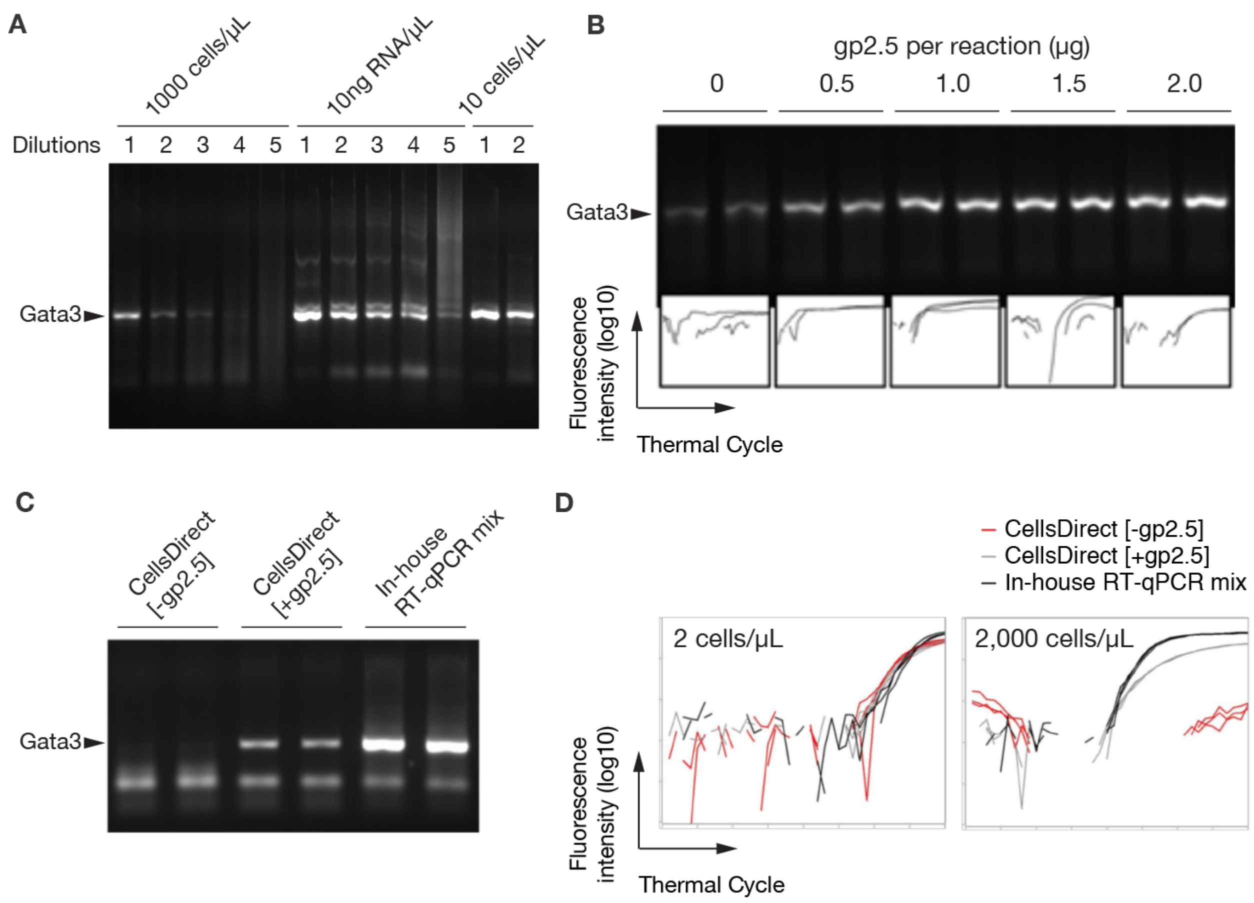

Figure S2. Inclusion of T7 bacteriophage single-stranded DNA binding protein, gene 2.5 protein (gp2.5) partially rescued qRT-PCR from lysate-mediate inhibition.

(A) Full-length gel electrophoresis image of Figure 2C. The same dilution factor was applied to samples labeled with the same number.

(B) Gata3 mRNA was analyzed in 10,000 EL4 cells using an in-house RT-qPCR mix with increasing gp2.5 concentrations. An agarose gel electrophoresis image visualized the increasing yield of Gata3 products as gp2.5 was increased from 0 μg to 2.0 μg per 10 μL reaction.

(C and D) Gata3 mRNA levels in 20 and 20,000 EL4 cells were analyzed using the in-house reaction, and CellsDirect kit with or without the addition of gp2.5 (10 μL reaction). A 2% agarose gel visualizes the end products of the RT-qPCR with 20,000 EL4 cells (C) and the qPCR amplification curves are shown in D.

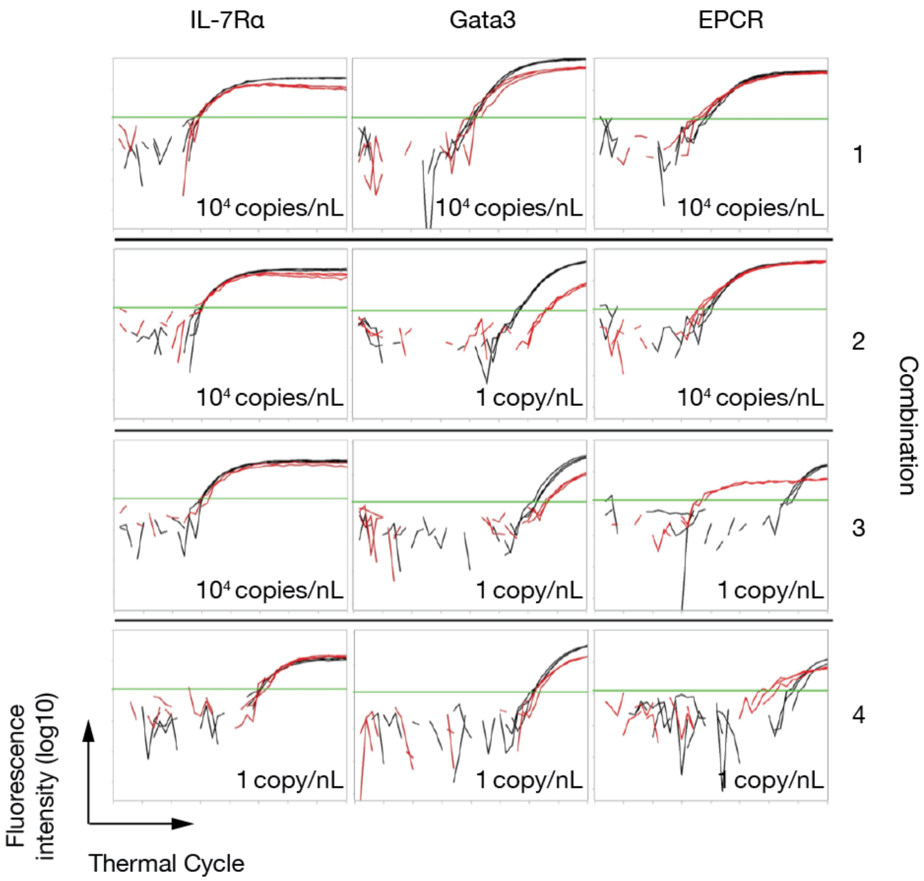

Figure S3. Triplex RT-qPCR in four combinations of high and low IVT abundances using our in-house singleplex RT-qPCR mix.

Combinations of IL-7Rα, Gata3 and EPCR IVTs were spiked into 10 µL RT-qPCRs to determine if the singleplex parameters could quantify vastly different target abundances. Genes analyzed in triplex reactions (red curves) were compared directly to their three respective singleplex controls (black curves) that were performed in the same experiment. Green lines indicate the thresholds for CT values.

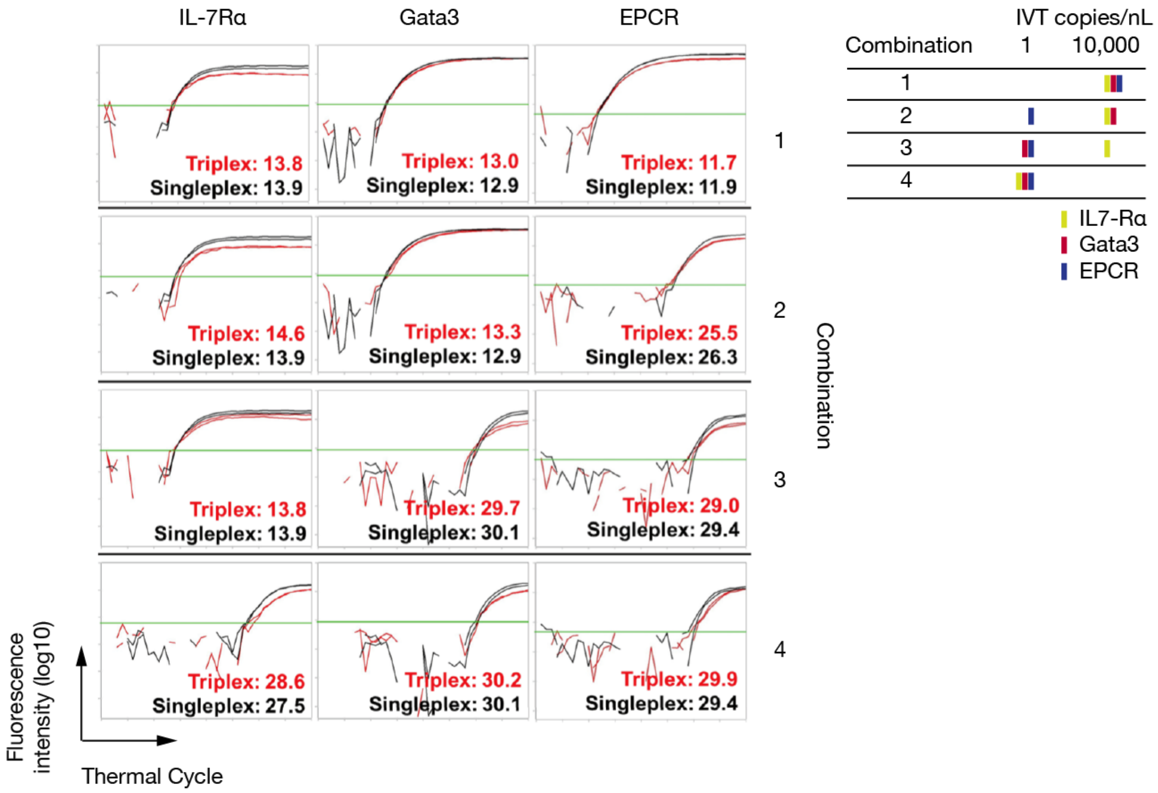

Figure S4. Quantification of IVT combinations 1–4 using reagents that have been adapted for a triplex RT-qPCR.

Four different combinations of IVT of the target genes were assembled, representing the extreme transcript abundance differences expected in single cells (right). These combinations were quantified using the optimized triplex RT-qPCR mix, and compared directly to the singleplex reactions. Thus, each triplex reaction was compared to three individual singleplex reactions. C_T values of each reaction is shown at the bottom right corner of its qPCR plot.

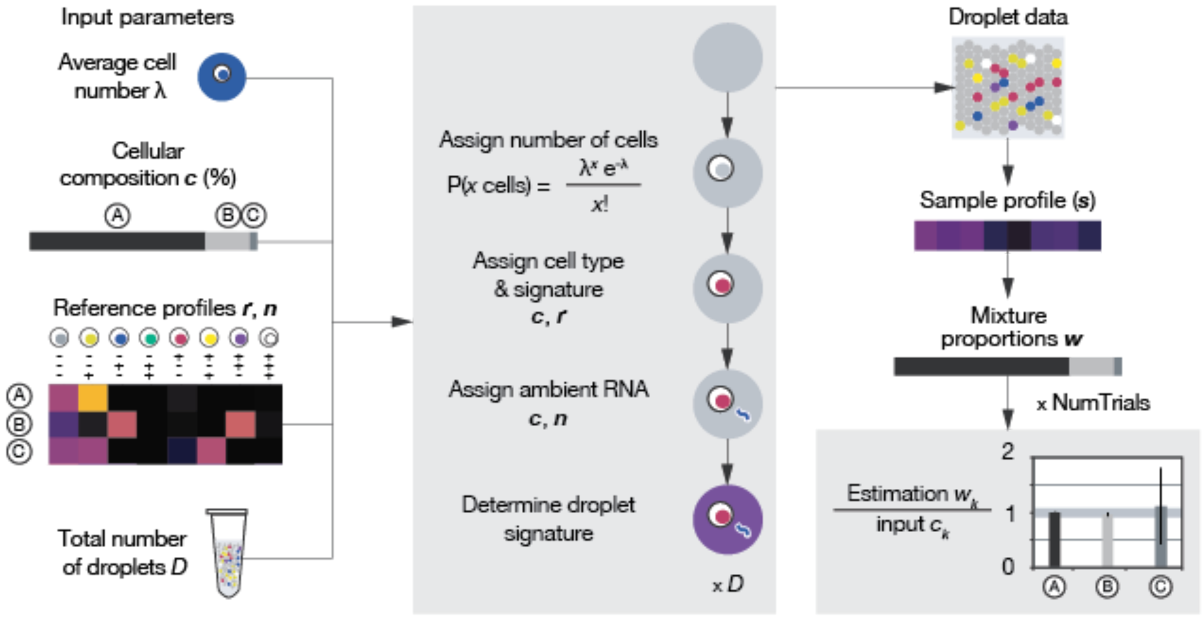

Figure S5. Schematic of the computer simulation.

The user input parameters that may affect the result of the assay, such as the average number of cells each droplet contains (λ) and the cellular composition of the sample mixture (***c***), into the simulation. The content and gene signature of each droplet in an experiment is then assigned randomly based on the input parameters, generating a simulated dataset. The simulated data is analyzed using our deconvolution model to produce a prediction ***w*** of the cellular composition of the sample. The experiment is repeated to generate a large number of predictions to evaluate their precision and the accuracy by comparing them to the input cellular composition ***c***.

Supplementary Tables

Table S1. Primers and Taqman probe design. T_m are shown in brackets.

Target gene	Amplicon Length (bp)	Primers	Taqman Probe	Fluorophore
Gata3<br>(Singleplex & multiplex)	196	F - 5' CAAGCTTCATAATATTAACAGACC (54 ºC)<br>R - 5' GCTGAAGGGAGAGATGTG (54C)	5’ AAGGCATCCAGACCCGAAAC (60 °C)	NED
EPCR (Singleplex)	95	F - 5’ GAACGTGTTCTTTCCTCTC (53°C)<br>R - 5’ CATCGAAGAAGACATGGG (52°C)	5' AGCCCTCCTCCTCCTCCTCC (64 ºC)	Jun
EPCR (Multiplex)	99	F - 5' GAACGTGTTCTTTCCTCTCAC (57 ºC)<br>R - 5' GCCACATCGAAGAAGACATG (57 ºC)		
IL-7Rα (Singleplex)	127	F - 5’ ACCAAAAGCAATGTATGAAATC (52°C)<br>R - 5’ CCATCCTCCTTGATTCTTG (53°C)	5' AAGGTAGAACTTGGACTCCACTC (66 ºC)	6-FAM
IL-7Rα (Multiplex)	87	F - 5' CATAACGATTACTTCAAAGGCT (54 ºC)<br>R - 5' CCATCCTCCTTGATTCTTG (53 ºC)		
Gusb	119	F - 5’ CTACTACACCCTTCCTATC (57 ºC)<br>R - 5’ CTCGGATATCTGAATCCTC (58C)	5' CAAGTTCCTCATAAACGGGAAGCC (66 ºC)	6-FAM

Table S2. In vitro transcript information and primer sequences used to generate cDNA template.

Target gene	Length (nt)	Molecular weight (g/mol)	Primer sequence
Gata3	733	235,639.5	F - 5' TAATACGACTCACTATAGGCAATGCCTGCGGACTCTACC
			R - 5' TTTTTTTTTTTTTTTTTTTTGAATGGCTTATTCACAAATGGGA
EPCR	729	235,309.1	F - 5' TAATACGACTCACTATAGGTCTCCTATTTCCAAGACAACCATC
			R - 5' TTTTTTTTTTTTTTTTTTTTGCTACGACCTTCCCTTACCAG
IL-7Rα	819	263,952.9	F - 5' TAATACGACTCACTATAGGCTCTTTTACGAGTGAAATGCC
			R - 5' TTTTTTTTTTTTTTTTTTTTTCTGTGTCCCTGTGTCTCCAAC

Table S3. Cost breakdown of multiplex droplet RT-PCR.

**Reagents**	**Cost per 1,000 cells in USD (10uL reaction with highly conservative estimate of 50% yield)**
TrisHCl	0.00001
KCl	0.00016
MgCl2	0.00043
RNase Inhibitor	0.53540
Taq polymerase (Invitrogen Taq DNA Polymerase, recombinant as reference for cost estimate)	2.06227
Binding protein	0.02955
dNTPs	0.03030
Primers	0.00178
Taqman probes	0.27510
NP-40	0.00000
Pluronic F68	0.00002
SuperScript III + DTT	0.03701
BSA	0.04727
Droplet Generation Oil	0.08685
PDMS	0.00345
**Total**	**3.10962**

Supplementary Materials and Methods

Microfluidic device fabrication

The droplet generator designs were modeled with AutoCAD (Autodesk) and printed on a transparency film (20000 dpi, Pacific Arts & Designs Inc.) as photomask. Photolithographic masters were prepared from SU-8 50 photoresist (Microchem) on silicon wafers (1190, Wafer World Inc.) based on the recommended protocol for 100 µm thickness by the manufacturer. The devices were fabricated using soft lithography. Poly(dimethylsiloxane) (PDMS) base and curing agent (Sylgard 184, Dow Corning) were mixed at a ratio of 10:1 by weight, degassed under vacuum, and poured onto the master placed in a Petri dish. The mixture was cured at 65 ºC in an oven for at least 4 hours. The molds were extracted with a scalpel and access holes were punched using a 0.75 mm biopsy punch. The devices were bonded to a blank piece of PDMS after being plasma-treated for 90 s at 600 mtorr using a Harrick Plasma Plasma Cleaner (PDC-001 and PDC-FMG). The sealed devices were baked at 65 ºC for at least 2 hr and hydrophobized by flowing Aquapel through the channels and then blowing them clear with air.

RNA purification and IVT

Primers used to generate the cDNA template for *in vitro* transcription were designed to flank the region targeted by the primers used in the multiplex reaction (Supplementary Table S2). IVTs are 700–800 bp segments of the mRNA transcript around the exon-exon boundaries targeted for subsequent RT-qPCR. The forward primers used to generate the cDNA template included a minimal T7 promoter sequence to allow the cDNA to be transcribed by T7 RNA polymerase, and the reverse primers contained a poly(T) sequence for the generation of transcripts with a poly(A) tail.

The generation of the cDNA by PCR was carried out in a 100 µL reaction consisting of 10 mM TrisHCl pH 8.3, 50 mM KCl, 1.5 mM MgCl2, 0.1 mg/mL BSA, 0.05% Triton X-100, 200 µM dNTPs, and a 250 nM pair of forward and reverse primers. The assembled reactions were heated to 94°C before 5 U of Taq polymerase were added. The addition of Taq polymerase at 94°C prevented the amplification of non-specific products at lower temperatures. All PCRs were performed on an Applied Biosystems 9700 instrument using the following thermal cycling conditions: 5 minutes at 94°C, followed by at least 25 cycles of 94°C for 15 seconds, 60°C annealing for 30 seconds and 72°C for 1 minute. Afterward, reactions were held at 72°C for 5 minutes, and then 4°C until they were loaded onto 2.0% agarose gels containing GelRed (Biotium) for analysis. The PCR products were then purified using the Qiagen QIAquick PCR

Purification Kit (column-based). The purified cDNA was quantified using a NanoDrop ND-1000, and stored at -20°C until it was needed for *in vitro* transcription.

In vitro transcription was performed using the MEGAscript T7 Transcription Kit (Invitrogen). 200 ng of purified cDNA was added to each reaction. The reactions were incubated at 37°C for 4 hours. 1 µL of DNase provided in the kit was added to digest the cDNA template, and the synthesized RNA was purified using TRIzol Reagent (Invitrogen). The purified transcripts were quantified using a Nanodrop ND-1000. IVT copy numbers were approximated based on molecular weight calculations performed using OligoCalc (Northwestern University). IVTs were diluted to 10^{10} copies, snap-frozen on dry ice, and stored at -80°C as 2–5 µL aliquots in PCR tubes for single use.
